# The Prognostic Role of Glycemia in Patients With Pancreatic Carcinoma: A Systematic Review and Meta-Analysis

**DOI:** 10.3389/fonc.2022.780909

**Published:** 2022-02-10

**Authors:** Xiaofang Wang, Wanfeng Xu, Xiaoru Hu, Xianghong Yang, Mingming Zhang

**Affiliations:** ^1^ Department of Clinical Oncology and Department of Hospice Care, Shengjing Hospital of China Medical University, Shenyang, China; ^2^ Department of Endocrinology, Shengjing Hospital of China Medical University, Shenyang, China; ^3^ Department of Pathology, Shengjing Hospital of China Medical University, Shenyang, China

**Keywords:** pancreatic neoplasms, glycemia, prognosis, complications, meta-analysis

## Abstract

**Background:**

Fasting blood glucose and glycated hemoglobin (HbA1c) levels are associated with the risk of pancreatic cancer.

**Aim:**

To examine the relationship between perioperative glucose and HbA1c levels and prognosis in patients with pancreatic cancer.

**Methods:**

PubMed, Embase, and the Cochrane Library were queried for potentially eligible studies published up to May 2021. The exposures were perioperative fasting glucose and HbA1c levels. The primary outcome was survival. The secondary outcome was complications. All analyses were performed using the random-effects model.

**Results:**

Ten studies (48,424 patients) were included. The pre-operative (HR=1.10, 95%CI: 0.89-1.35; I^2^ = 45.1%, P_heterogeneity_=0.078) and postoperative (HR=1.19, 95%CI: 0.92-1.54; I^2^ = 67.9%, P_heterogeneity_=0.001) blood glucose levels were not associated with the survival to pancreatic cancer. Similar results were observed for HbA1c (HR=1.09, 95%CI: 0.75-1.58; I^2 ^= 64.2%, P_heterogeneity_=0.039), fasting blood glucose (FBG)/HbA1c (HR=1.16, 95%CI: 0.67-1.68; I^2 ^= 0.0%, P_heterogeneity_=0.928), and FBG (HR=1.75, 95%CI: 0.81-3.75; I^2 ^= 79.4%, P_heterogeneity_=0.008). Pre-operative blood glucose levels were not associated with postoperative complications (OR=0.90, 95%CI: 0.52-1.56), but postoperative glucose levels were associated with postoperative complications (OR=3.06, 95%CI: 1.88-4.97; I^2 ^= 0.0%, P_heterogeneity_=0.619).

**Conclusion:**

Blood glucose, FBG, and HbA1c levels are not associated with the survival of patients with pancreatic cancer. Postoperative blood glucose levels could predict postoperative complications.

## Introduction

Pancreatic cancer has an age-standardized incidence of 5.5 per 100,000 men (ranks tenth worldwide) and 4 per 100,000 women (ranks ninth) (1, 2). It is the ninth most common cause of cancer death worldwide (432,242 deaths in 2018) (1, 2) and, because it is usually diagnosed in its late stages, one of the most fatal cancers with a 5-year survival rate of <5% (2). It usually affects people >55 years of age (3–5). The risk factors for pancreatic cancer include smoking, genetic predispositions, family history, obesity, chronic pancreatitis, and diabetes (3–6). The management of pancreatic cancer is multidisciplinary, but it often presents at advanced stages. Surgical resection should be offered to patients with no clinical evidence of metastases, performance status and comorbidity profile suitable for resection, no radiographic interface between primary tumor and mesenteric vasculature on highly defined cross-sectional imaging, cancer antigen (CA) 19-9 level (in patients without jaundice) suggestive of potentially curable disease (7–9). Neoadjuvant therapy can be offered to patients in whom surgical resection is planned, and in the presence of suspected (but not diagnostic) radiographic findings of extrapancreatic disease, performance status, and comorbidity profile currently not suitable for upfront resection, but is potentially reversible, a radiographic interface between primary tumor and mesenteric vasculature on cross-sectional imaging that does not meet criteria for upfront resection, and CA 19-9 level (in patients without jaundice) suggestive of disseminated disease (7–9). Still, the 5-year overall survival (OS) is poor, at <10% (3–5).

Diabetes is both a risk factor and a complication of pancreatic cancer (3–6). Indeed, long-term diabetes is associated with an increased risk of pancreatic cancer, and early-stage pancreatic cancer was reported to cause new-onset diabetes, but the pathogenesis is unclear (3–6). Previous studies showed that long-term diabetes (≥2 years) is associated with increased risk of pancreatic cancer (10), increased fasting blood glucose might be associated with an increased risk of pancreatic cancer (11), and even prediabetes is associated with an increased risk of pancreatic cancer (12). Conditions associated with diabetes are also associated with pancreatic cancer, including obesity (13–15) and low physical activity (16).

A linear dose-response relationship between fasting blood glucose levels and pancreatic cancer risk has been reported in a meta-analysis (11). A similar relationship was observed in the Asian population in pooled analyses (17, 18). A systematic review reported an association between glycosylated hemoglobin (HbA1c) and pancreatic cancer (19), which is probable since HbA1c levels reflect glycemic control over the past 3 months (20). It is, therefore, possible that random blood glucose levels, fasting glucose levels, and HbA1c might be associated with the prognosis of pancreatic cancer.

Therefore, the meta-analysis aimed to examine the relationship between perioperative glucose and HbA1c levels and prognosis in patients with pancreatic cancer. The results could provide a simple and easy method to identify patients at higher risk of poor outcomes.

## Methods

### Literature Search

The systematic review and meta-analysis was performed according to the Preferred Reporting Items for Systematic Reviews and Meta-Analyses (PRISMA) guidelines (21). The study was designed using the PICOS principle (22). PubMed, Embase, and the Cochrane Library were queried for potentially eligible studies published up to May 2021 using the MeSH terms of ‘Pancreatic neoplasms’, ‘blood glucose’, and ‘Glycosylated Hemoglobin A’, as well as relevant key words, followed by screening based on the inclusion and exclusion criteria by two investigators (** and **), independently. Disagreements were solved by discussion.

### Eligibility Criteria

The inclusion criteria were 1) patients: adults (>18 years of age) with a diagnosis of pancreatic cancer, 2) exposure: low/high levels of blood glucose and HbA1c according to the cut-off values of fasting blood glucose and HbA1c in each study, 3) primary outcome: reported hazards ratios (HRs) with their 95% confidence interval (95% CIs) for survival outcomes, 4) secondary outcome: complications, 5) study type: prognosis study, and 6) full-text in English. The eligible reports were published from database inception up to May 2021. The exclusion criteria were 1) letters to the editor, case report, protocol, review, meta-analysis, *in vivo* study, or *in vitro* study, 2) full-text not available, or 3) no data could be extracted.

### Data Extraction

Data were extracted by two investigators according to a pre-specified protocol: study characteristics (authors, year of publication, country, study design, and sample size), patient’s characteristics (age and sex), exposure parameters (measurement timing, cut-off value of blood glucose, and type of exposure), and outcomes (survival and complications).

### Quality of the Evidence

The level of evidence of all articles was assessed independently by two authors according to the Newcastle-Ottawa Scale (NOS) criteria for the quality assessment of cohort studies (23) and the National Institutes of Health (NIH) criteria for case series (24). Discrepancies in the assessment were resolved through discussion until a consensus was reached.

### Statistical Analysis

All analyses were performed using STATA SE 14.0 (StataCorp, College Station, TX, USA). Time-to-event data were summarized as hazard ratio HRs and 95%CIs confidence interval (95%CI). Dichotomous variables were summarized as odds ratios (ORs) and 95%CIs. Statistical heterogeneity among the studies was calculated using Cochran’s Q-test and the I^2^ index. An I^2^ >50% and Q-test P<0.10 were considered suggestive of high heterogeneity. Once heterogeneity was noticed, the source of heterogeneity was investigated using subgroup analyses by stratifying the original estimates according to the study characteristics. The meta-analysis was performed using a random-effects model. P-values <0.05 were considered statistically significant. The potential publication bias was assessed by the visual inspection of funnel plots.

## Results

### Literature Search

**Figure 1:**
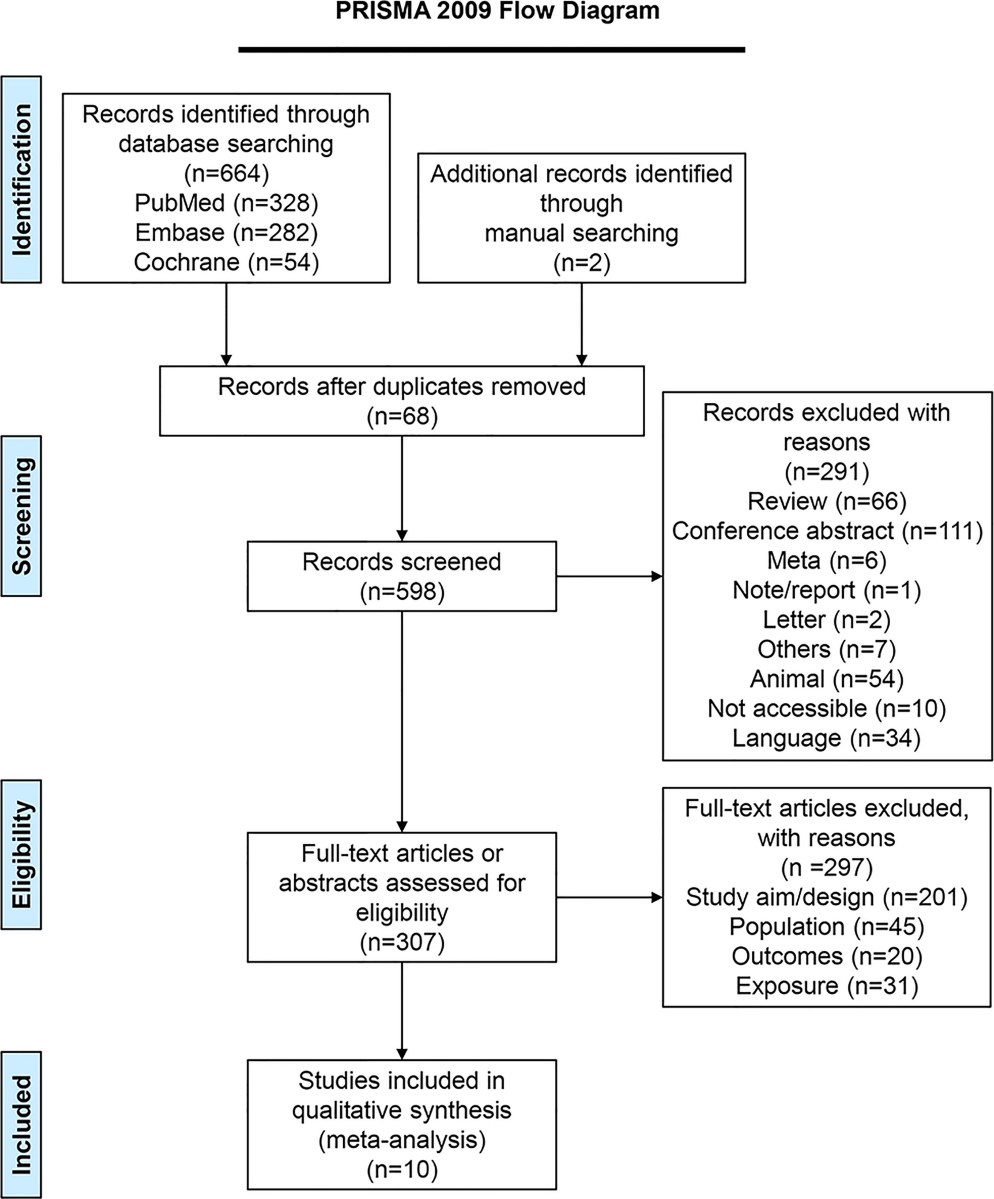
PRISMA 2009 Flow Diagram.

**Figure 1** presents the literature search process. The initial search yielded 664 records, and another two articles were retrieved manually. Sixty-eight duplicates were removed, and 598 records were screened. After excluding 291 records, 307 full-text articles were assessed for eligibility, and 297 were excluded (study aim/design, n=201; population, n=45; outcomes, n=20, exposure, n=31). Finally, ten studies (25–34) were included.

### Characteristics of the Studies

**Table 1** presents the characteristics of the studies. There were two prospective observational studies, four retrospective observational studies, two retrospective cohort studies, one pooled analysis, and one case series. Four studies were from North America, three were from Europe, and three were from China. The meta-analysis included 48,424 patients.

**Table 1:** Characteristics of the included studies.

Study	Design	Country	Sample size	Age, years (mean or median)	Sex, male %	Measurement timing	Cut-off value	Type of exposure
Alpertunga 2021 (25)	Prospective observational study	USA	73	72 (44–90)	52.1	Pre-/post-treatment	pre: ≤140 mg/dl	HbA1c
							post: ≤120 mg/dl	
Eshuis 2011 (26)	Retrospective observational study	Netherlands	330	62.3 (12)	56	Pre-/post-treatment	pre: 135 mg/dl	FBG
							post: 142 mg/dl	
Fan 2014 (27)	Prospective observational study	USA	240	67	52.5	Pre-treatment	≥6.5%	HbA1c
Gong 2020 (28)	Retrospective cohort study	China	246	/	62	Pre-treatment	>5.6 mmol/L	FBG
Iarrobino 2019 (29)	Retrospective cohort study	USA	303	70 (33–90)	50.2	Pre-treatment	≥200 mg/dL	HbA1c
Nagai 2017 (30)	Pooled analysis	Switzerland	46387	57.7 (10.4)	44	Post-treatment	90-96 mg/dL	FBG
							97-109 mg/dL	
							110-125 mg/dL	
							≥126 mg/dL	
Rajamanickam 2016 (31)	Retrospective observational study	USA	123	66 (16)	51	Pre-treatment	>6.5%	HbA1c
Sandini 2019 (32)	Retrospective observational study	Germany	417	58 (48–66)	56.1	Pre-treatment	FBG >126 mg/dL; HbA1c >6.5%	FBG/HbA1c
Shi 2017 (33)	Case series	China	52	60.3 (9)	61.5	Pre-/post-treatment	≥7%; >110 mg/dL	FBG/HbA1c
Zhang 2021 (34)	Retrospective observational study	China	253	60 (37–83)	58.5	Pre-treatment	>6.11 mmol/L	FBG

Among the cohort studies, two scored 6 points on the NOS, four scored 7 points, and three scored 8 points (**Supplementary Table S1**). The case series was deemed of good quality (**Supplementary Table S2**).

### Association Between Glycemic Indexes and Survival to Pancreatic Cancer

**Figure 2:**
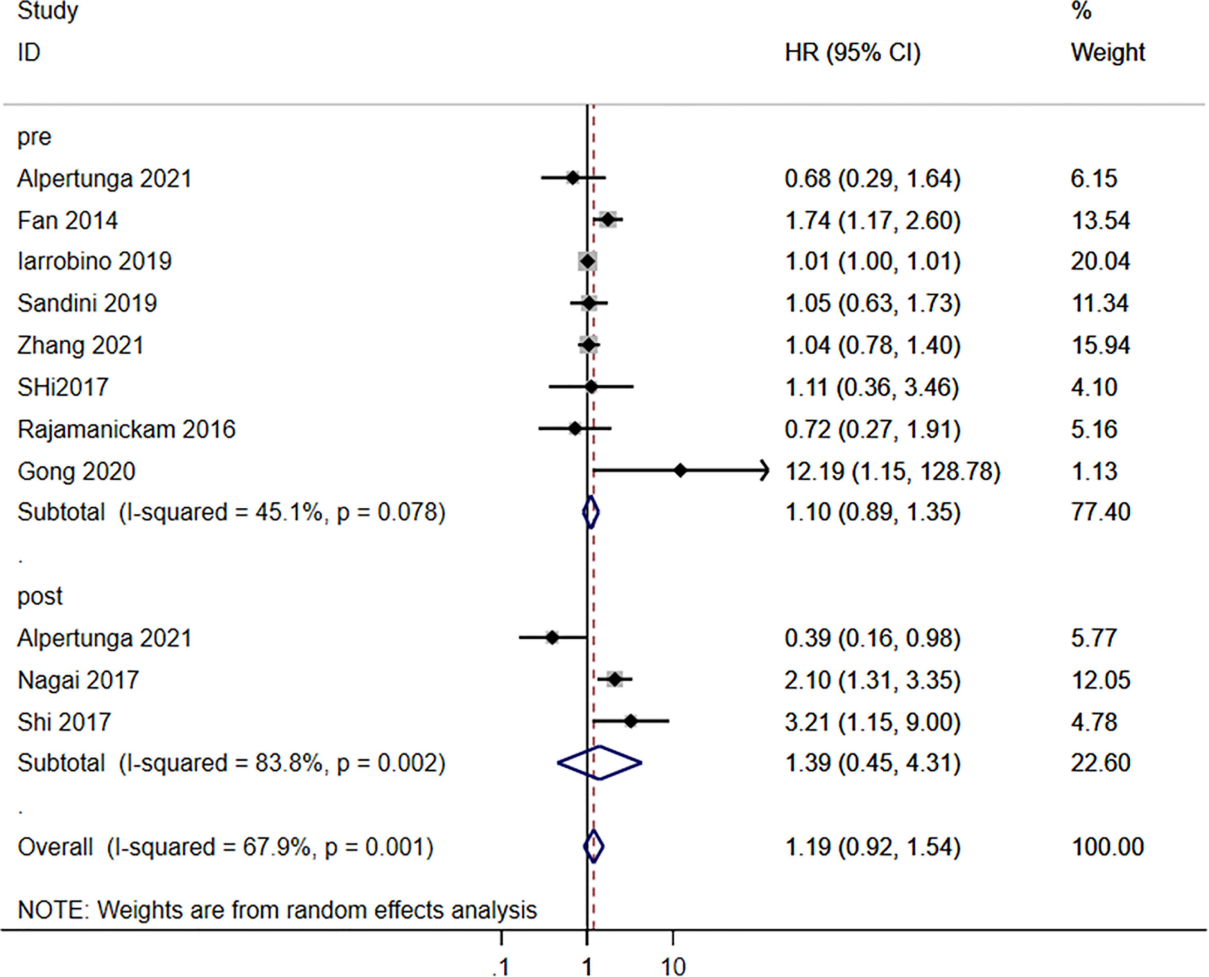
Subgroup analysis of overall survival in the pancreatic cancer population according to the blood glucose levels according to pre- and postoperative.

**Figure 3:**
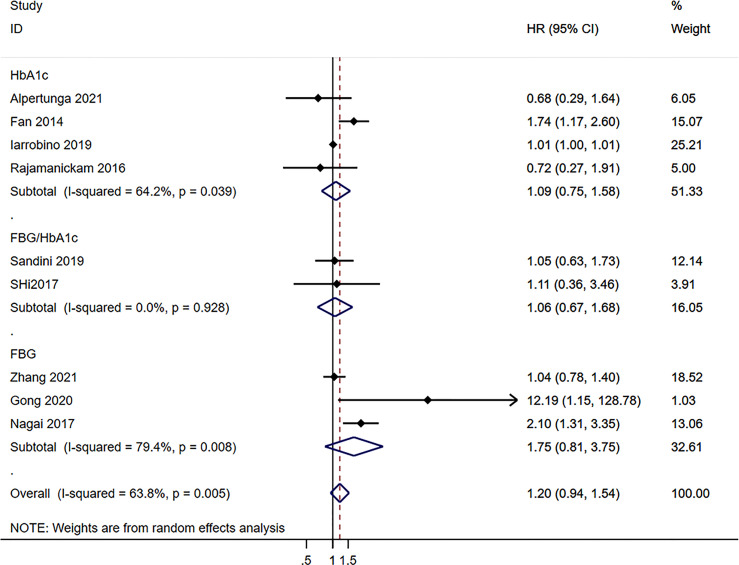
Subgroup analysis of overall survival in the pancreatic cancer population according to the blood glucose levels according to the type of exposure.

Neither the pre-operative (HR=1.10, 95%CI: 0.89-1.35; I^2^ = 45.1%, P_heterogeneity_=0.078) nor the postoperative (HR=1.19, 95%CI: 0.92-1.54; I^2^ = 67.9%, P_heterogeneity_=0.001) blood glucose levels were associated with the survival to pancreatic cancer, for an overall HR=1.19 (95%CI: 0.92-1.54; I^2^ = 67.9%, P_heterogeneity_=0.001) (**Figure 2**). HbA1c (HR=1.09, 95%CI: 0.75-1.58; I^2^ = 64.2%, P_heterogeneity_=0.039), fasting blood glucose (FBG)/HbA1c (HR=1.16, 95%CI: 0.67-1.68; I^2^ = 0.0%, P_heterogeneity_=0.928), and FBG (HR=1.75, 95%CI: 0.81-3.75; I^2^ = 79.4%, P_heterogeneity_=0.008) were not associated with the survival to pancreatic cancer, for an overall HR=1.20 (95%CI: 0.94-1.54; I^2^ = 63.8%, P_heterogeneity_=0.005) (**Figure 3**).

### Postoperative Complications

**Figure 4:**
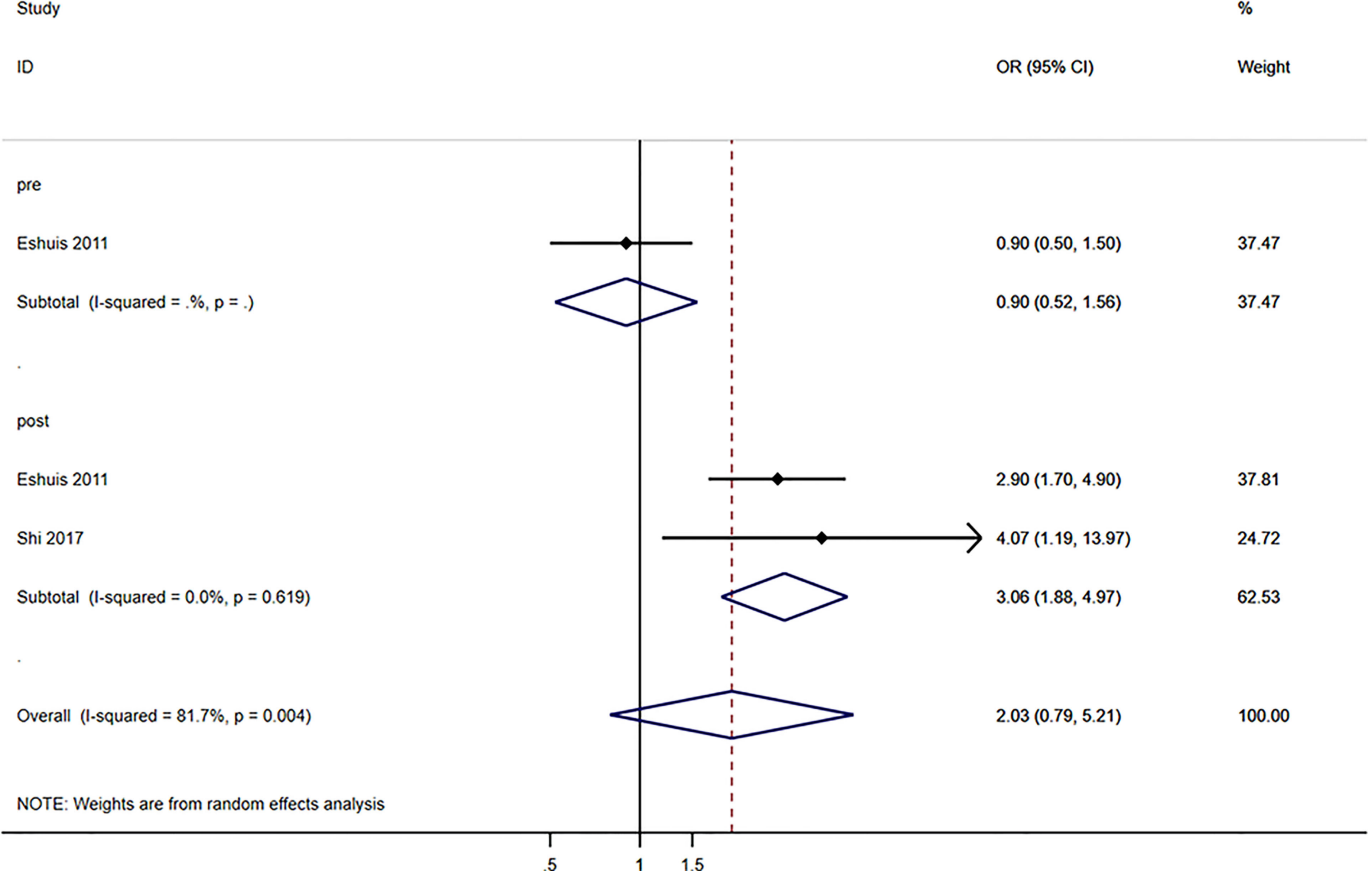
The prognostic role of the blood glucose values in postoperative complications.

Globally, blood glucose levels were not associated with postoperative complications (OR=2.03, 95%CI: 0.79-5.21, I^2^ = 81.7%, P_heterogeneity_=0.004) (**Figure 4**). When considering the timing of blood glucose measurement, pre-operative blood glucose levels were not associated with postoperative complications (OR=0.90, 95%CI: 0.52-1.56), but postoperative glucose levels were associated with postoperative complications (OR=3.06, 95%CI: 1.88-4.97; I^2^ = 0.0%, P_heterogeneity_=0.619) (**Figure 4**).

### Publication Bias

**Figure 5:**
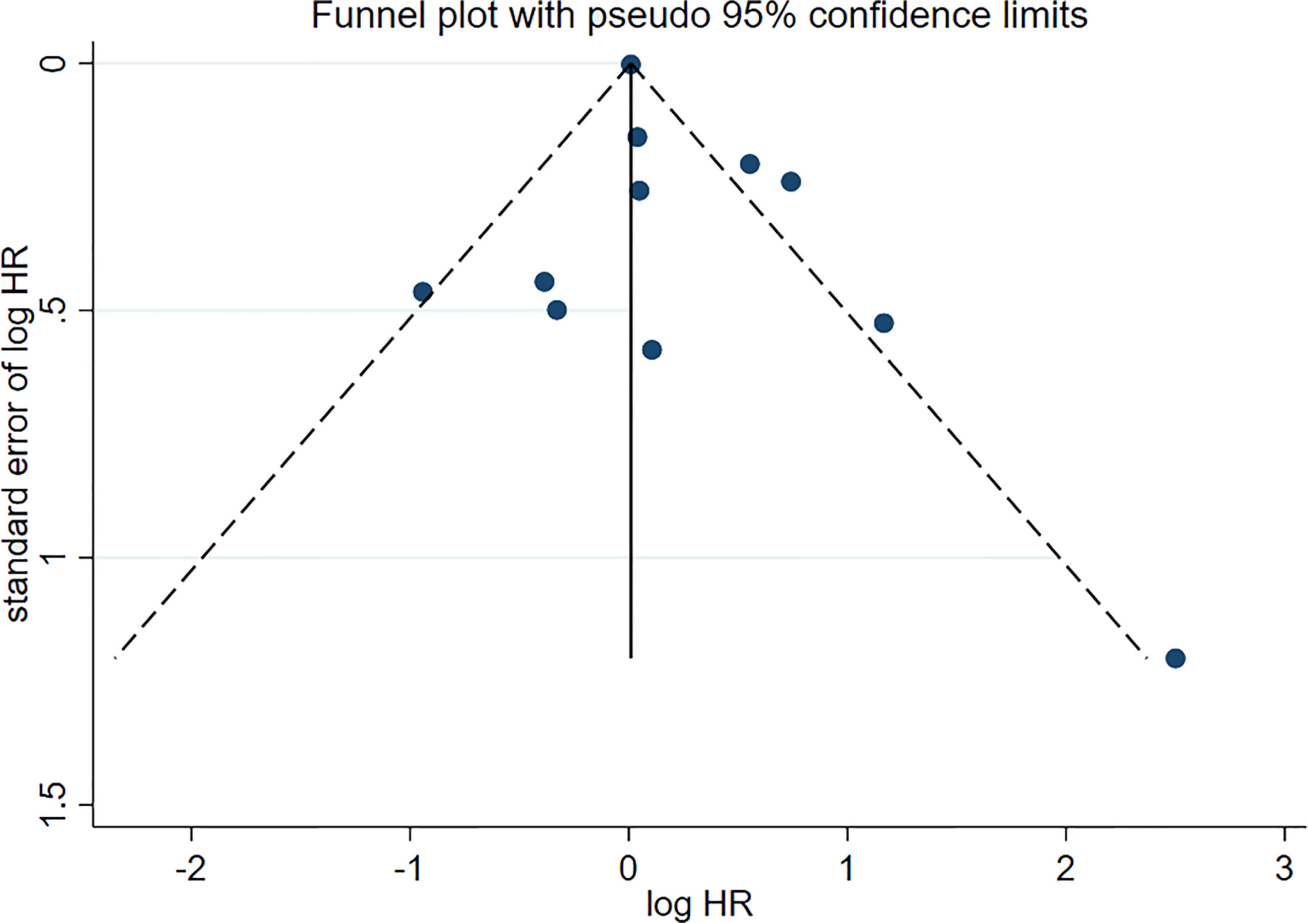
Funnel plot of publication bias.

No asymmetry was found in the funnel plot (**Figure 5**). Therefore, the publication bias did not affect the outcome.

### Sensitivity Analysis

**Figure 6:**
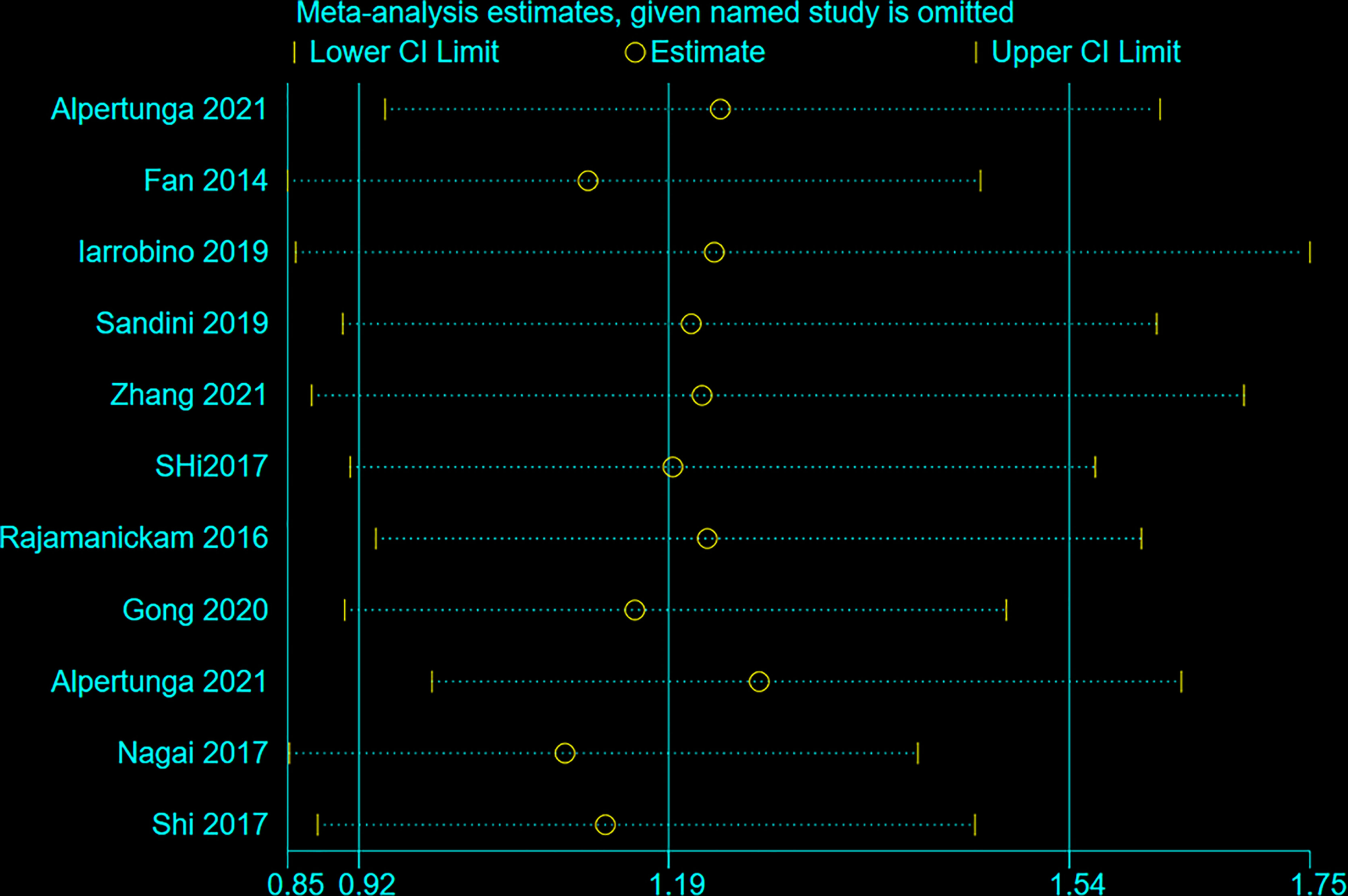
Sensitivity analysis of overall survival according to blood glucose value.

**Figure 6** shows that no individual study influenced the meta-analysis. Hence, the results were robust.

## Discussion

Fasting blood glucose and HbA1c levels are associated with the risk of pancreatic cancer (11, 17–19). This meta-analysis aimed to examine the relationship between perioperative glucose and HbA1c levels and prognosis in patients with pancreatic cancer. The results suggest that blood glucose, FBG, and HbA1c levels are not associated with the survival of patients with pancreatic cancer. Postoperative blood glucose levels could predict postoperative complications.

Previous studies and meta-analyses showed that glucose and HbA1c levels were associated with the occurrence of pancreatic cancer (11, 17–19). Indeed, diabetes or impaired glucose metabolism is both a risk factor and a complication of pancreatic cancer (3–6). Still, the pathogenesis of glucose dysmetabolism in pancreatic cancer is unknown (3–6). Considering the association between elevated glucose levels and the development of pancreatic cancer, the present meta-analysis hypothesized that blood glucose levels could also be associated with the prognosis of pancreatic cancer. The present meta-analysis suggests that blood glucose, FBG, and HbA1c levels are not associated with the prognosis of pancreatic cancer. It is supported by a previous meta-analysis that showed that HbA1c levels were not associated with the prognosis of colorectal, breast, bladder, pancreatic, or prostate cancer (35). Still, conflicting results can be found in the literature. Indeed, Cheon et al. (36) showed that HbA1c levels were associated with the prognosis of pancreatic cancer and that metformin improved the prognosis. Gapstur et al. (37) showed that after adjustment for age, race, smoking, and body mass index, the risk of pancreatic cancer mortality increased with the postload plasma glucose levels; still, they examined pancreatic cancer mortality at the population-based level and not specifically in the cancer patients.

High glucose levels are associated with several pathophysiological events that are key for cancer progression. Chronic hyperinsulinemia in insulin-resistant patients increases IGF-I bioavailability and hence will increase cell growth and affect the levels of various hormones to induce a cancer-promoting environment (38). In addition, angiopoietin (Ang) is an important pro-angiogenic factor that regulates angiogenesis (39). Serum Ang-2 levels are closely related to the severity of diabetes (40). High concentrations of VEGF in the tumor microenvironment promote Ang-2 as angiogenesis (39). Moreover, Ang-2 is a potential surrogate marker of metastasis in patients with metastatic pancreatic cancer (41). Targeting diabetes and its dysregulated hormones might be a way to manage the risk of cancer (42). Still, diabetes and cancer share some risk factors, like obesity, age, diet, and physical activity (43).

Pancreatic stump closure is an important issue in the resection of pancreatic cancer and is associated with the risk of pancreatic fistula, delayed gastric emptying, and pancreaticojejunostomy leakage (26, 33, 44, 45). Such complications can be life-threatening in the short term. Glucose levels are a punctual measurement of glucose metabolism, while HbAc1 levels represent glucose control over the past 2-3 months (46). Therefore, blood glucose levels vary widely according to meals, waking state, and physical activity. Even in the fasting state, glucose levels can vary according to trauma, comorbidities, and the general condition of a patient. Of note, after trauma (including surgical trauma), blood glucose levels are elevated in response to the activated sympathoadrenal system, hypothalamus, and pituitary gland, leading to increased secretion of catecholamines and glucocorticoids, in turn leading to hyperglycemia (47). In the present meta-analysis, only the postoperative glucose levels were associated with postoperative complications. The complications included infections complications, complications Dindo-Clavien IIIa or higher, delayed gastric emptying, pancreaticojejunostomy leakage, and relaparotomy (26, 33). On the other hand, the included studies did not always report the exact complications (e.g., Dindo-Clavien IIIa complications can include many different complications). Further study is necessary to examine the exact complications associated with high glucose levels after pancreatic cancer resection. Still, the results suggest that the postoperative glucose levels could be monitored to screen for patients at a higher risk of complications.

Heterogeneity in a meta-analysis is an important source of bias that can affect the results. Heterogeneity represents the effect of the differences among the included studies. In the present meta-analysis, an important source of heterogeneity is the country. Indeed, the United States of America has high proportions of obesity and type 2 diabetes, which affects blood glucose levels. Other sources of bias could be the age of the patients and the proportion of males. Still, the most important source of bias in the present meta-analysis is the use of different cut-off values to determine high glucose or high HbA1c. Of course, the use of different cut-offs will directly affect the association with the outcome. It highlights the need for standardized cut-off values for future trials and studies.

The strength of this meta-analysis is that it examined the most commonly assessed glucose control indexes. Still, this study has limitations. A meta-analysis inherits the limits of all the included studies, and caution must be applied while extrapolating the results. Most of the included studies were retrospective studies with relatively small cohorts of patients. Patient data were acquired retrospectively, and unavoidable confounding is possible. Second, for studies that used FBG as the exposure factor, the FBG levels of patients may fluctuate due to multiple factors, and one FBG test may not accurately reflect the blood glucose levels. Parameters like glycated hemoglobin (HbA1c) can better reflect long-term glycemic levels (20). Third, the regimens of adjuvant chemotherapy and types of surgery for each patient were not specified in most of the included studies. Therefore, we cannot analyze such data. Fourth, the studies that only reported pre-operative blood glucose levels might threaten the validity of our results. In addition, Fan et al. (27) only obtained HbA1c levels at each patient’s initial visits to the pancreatic clinical center. It would be worthwhile to obtain HbA1c levels at regular intervals both during cancer treatment and at regular post-treatment follow-up visits to elucidate further the role played by glycemic control in improving survival in pancreatic cancer patients. Finally, study selection will influence the conclusions of a meta-analysis, and a rigorous process must be followed to ensure the validity and robustness of the conclusions. Indeed, combining studies will increase the sample size and thus the power of the exposure of interest. Still, publication bias is possible since positive studies are more likely to be published. Even in the absence of publication bias, a faulty search can miss some studies. This meta-analysis used a standardized search string. The search was performed independently by two authors. Then, their results were compared. Any discrepancy (e.g., a study was selected by one author but not by the other) was solved by discussion until a consensus was reached. The meta-analysis suggested that the postoperative glucose levels were associated with postoperative complications. It could be monitored to screen for patients at a higher risk of complications. Considering the included studies did not always report the exact complications. Further study is necessary to examine the exact complications associated with high glucose levels after pancreatic cancer resection.

In conclusion, blood glucose, FBG, and HbA1c levels are not associated with the survival of patients with pancreatic cancer. Postoperative blood glucose levels could predict postoperative complications.

To examine the relationship between perioperative glucose and glycated hemoglobin (HbA1c) levels and prognosis in patients with pancreatic cancer. Ten studies (48,424 patients) were included in final analysis. The pre-operative (HR=1.10, 95% CI: 0.89-1.35; I2 = 45.1%, Pheterogeneity=0.078) and postoperative (HR=1.19, 95%CI: 0.92- 1.54; I2 = 67.9%, Pheterogeneity=0.001) blood glucose levels were not associated with the survival to pancreatic cancer. Similar results were observed for HbA1c (HR=1.09, 95%CI: 0.75-1.58; I2 = 64.2%, Pheterogeneity=0.039), fasting blood glucose (FBG)/HbA1c (HR=1.16, 95%CI: 0.67-1.68; I2 = 0.0%, Pheterogeneity=0.928), and FBG (HR=1.75, 95% CI: 0.81-3.75; I2 = 79.4%, Pheterogeneity=0.008). Pre-operative blood glucose levels were not associated with postoperative complications (OR=0.90, 95%CI: 0.52-1.56), but postoperative glucose levels were associated with postoperative complications (OR=3.06, 95%CI: 1.88-4.97; I2 = 0.0%, Pheterogeneity=0.619). In conclusion, blood glucose, FBG, and HbA1c levels are not associated with the survival of patients with pancreatic cancer. Postoperative blood glucose levels could predict postoperative complications.

## Data Availability Statement

The original contributions presented in the study are included in the article/**Supplementary Material**. Further inquiries can be directed to the corresponding author.

## Author Contributions

Conceptualization: MZ. Methodology: XW. Software: XW. Formal analysis: XW. Investigation: XH. Resources: WX. Data curation: MZ. Writing—original draft preparation: XW. Writing—review and editing: MZ. Supervision: MZ. Project administration: MZ. Funding acquisition: XY. All authors have read and agreed to the published version of the manuscript.

## Funding

This study was funded by the National Natural Science Foundation of China (Grant No. 81773108).

## Conflict of Interest

The authors declare that the research was conducted in the absence of any commercial or financial relationships that could be construed as a potential conflict of interest.

## Publisher’s Note

All claims expressed in this article are solely those of the authors and do not necessarily represent those of their affiliated organizations, or those of the publisher, the editors and the reviewers. Any product that may be evaluated in this article, or claim that may be made by its manufacturer, is not guaranteed or endorsed by the publisher.
