# Supplementary material for: The Prognostic Role of Glycemia in Patients With Pancreatic Carcinoma: A Systematic Review and Meta-Analysis
Source: Front Oncol. 2022 Feb 10;12:780909. doi: 10.3389/fonc.2022.780909 (PMC8866248; doi:10.3389/fonc.2022.780909)
Supplement: Supplementary file 1 [file Table_1.docx]

**Supplementary Table S1.** NOS criteria for cohort studies

| Study | Representativeness of the exposed cohort | Selection of the non-exposed cohort | Ascertainment of exposure | Demonstration that outcome of interest was not present at the start of the study | Comparability of cohorts based on the design or analysis | Assessment of outcome | Was follow-up long enough for outcomes to occur | Adequacy of follow up of cohorts | Total quality scores |
| --- | --- | --- | --- | --- | --- | --- | --- | --- | --- |
| Alpertunga 2021 [22] | ⭐ | ⭐ | ⭐ | ⭐ | ⭐⭐ | ⭐ | ⭐ | / | 8 |
| Eshuis 2011 [23] | ⭐ | ⭐ | ⭐ | / | ⭐ | ⭐ | ⭐ | / | 6 |
| Fan 2014 [24] | ⭐ | ⭐ | ⭐ | ⭐ | ⭐ | ⭐ | ⭐ | ⭐ | 8 |
| Gong 2020 [25] | ⭐ | ⭐ | ⭐ | / | ⭐⭐ | ⭐ | / | / | 6 |
| Iarrobino 2019 [26] | ⭐ | ⭐ | ⭐ | / | ⭐ | ⭐ | ⭐ | ⭐ | 7 |
| Nagai 2017[27] | ⭐ | ⭐ | ⭐ | / | ⭐ | ⭐ | ⭐ | ⭐ | 7 |
| Rajamanickam 2016 [28] | ⭐ | ⭐ | ⭐ | / | ⭐⭐ | ⭐ | ⭐ | / | 7 |
| Sandini 2019[29] | ⭐ | ⭐ | ⭐ | / | ⭐ | ⭐ | ⭐ | ⭐ | 7 |
| Zhang 2021[31] | ⭐ | ⭐ | ⭐ | / | ⭐⭐ | ⭐ | ⭐ | ⭐ | 8 |

**Supplementary Table S2.** NIH quality assessment for case series

| Study | Was the study question or objective clearly stated? | Was the study population clearly and fully described, including a case definition? | Were the cases consecutive? | Were the subjects comparable? | Was the intervention clearly described? | Was the length of follow-up adequate? | Were the statistical methods well-described? | Were the results well-described? | Quality rating | |
| --- | --- | --- | --- | --- | --- | --- | --- | --- | --- | --- |
|  |  |  |  |  |  |  |  |  | Rater #1 | Rater #2 |
| Shi 2016 [30] | Y | Y | CD | Y | Y | Y | Y | Y | Good | Good |

Abbreviation: CD, cannot determine; NA, not applicable; NR, not reported; Y, yes; N, No
